# Extramedullary myeloma progressed to plasmablastic myeloma

**DOI:** 10.46989/001c.92050

**Published:** 2024-01-12

**Authors:** Ke Xu, Charalampia Kyriakou

**Affiliations:** 1 Haematology University College London Hospitals NHS Foundation Trust; 2 Specialist Integrated Haematology Malignancy Diagnostic Service, Health Services Laboratories University College London Hospitals NHS Foundation Trust

**Keywords:** Plasmablastic myeloma, extramedullary myeloma

Dear Editor,

A 55-year-old man was diagnosed with IgA Lambda bone-related extramedullary myeloma. Bone marrow trephine sample showed less than 5% plasma cells with polytypic light chain staining. Target CD138-cell fluorescence *in situ* hybridization (FISH) showed normal signal for 1q, 17p and loss of one copy of IGH in 11% of cells analysed. Paraprotein was 27g/L. PET/CT (Figure 1.A) showed FDG-avid disease at right scapula, T4 and humerus. He was treated with bortezomib, cyclophosphamide and dexamethasone, followed by high-dose melphalan and autologous transplantation. He achieved a very good partial response (VGPR) (paraprotein was undetectable by serum protein electrophoresis but detectable by immunofixation, and PET/CT showed complete remission), followed by lenalidomide maintenance. One year later, he relapsed with large left chest wall mass, liver and bowel involvement (Figure 1.B), and paraprotein 8g/L. Biopsy of the chest wall mass confirmed neoplastic plasma cells, with no evidence of plasmablasts. Bone marrow trephine sample showed 3% plasma cells. Target CD138-cell FISH showed 17p deletion in 40% of cells analysed, indicating around 1% neoplastic plasma cells presented in the bone marrow sample. His disease progressed to more aggressive bone-independent extramedullary myeloma. He was treated with DT-PACE (dexamethasone, thalidomide, cisplatin, etoposide, cyclophosphamide, doxorubicin) to VGPR, followed by weekly bortezomib and dexamethasone. Ten months later he relapsed again, with paraprotein 47g/L and progressive pancytopenia (Hb 94g/L, WBC 8.98 x10^9^/L, platelet 7 x10^9^/L). PET/CT showed recurrent large left chest wall mass and widespread disease (Figure 1.C). Bone marrow analysis showed 95% plasmablasts with large nuclei, fine chromatin, prominent nucleoli and cytoplasmic vacuolation (Figure 1.D). Flow cytometry showed these cells were positive for CD138, CD38, CD79b, and CD56, and negative for CD34, CD117, CD19, and CD20. Target CD138-cell FISH showed 1q gain (3 copies) in 92% of cells analyzed and 17p deletion in 92% of cells analyzed. Next-generation sequencing (Archer Variantplex, Archer, Boulder, CO, USA) identified *BRAF* p.Val600Glu (variant allele frequency (VAF) 65%) variant. Molecular karyotyping (using 8x60K oligonucleotide arrays, Agilent) identified a complex genome with losses at 2p; 16q22/q24; 22q11/q13 and 17p13/p11; gains at 1q21/q44; 3q11/q29; 6p25/p21; 7p22/p11; 7q11/q36, as well as trisomy of chromosomes 19 and 20. He received radiotherapy for the chest wall mass and systemic chemotherapy with isatuximab, pomalidomide, and dexamethasone. He passed away two months later with refractory myeloma.

**Figure 1.**
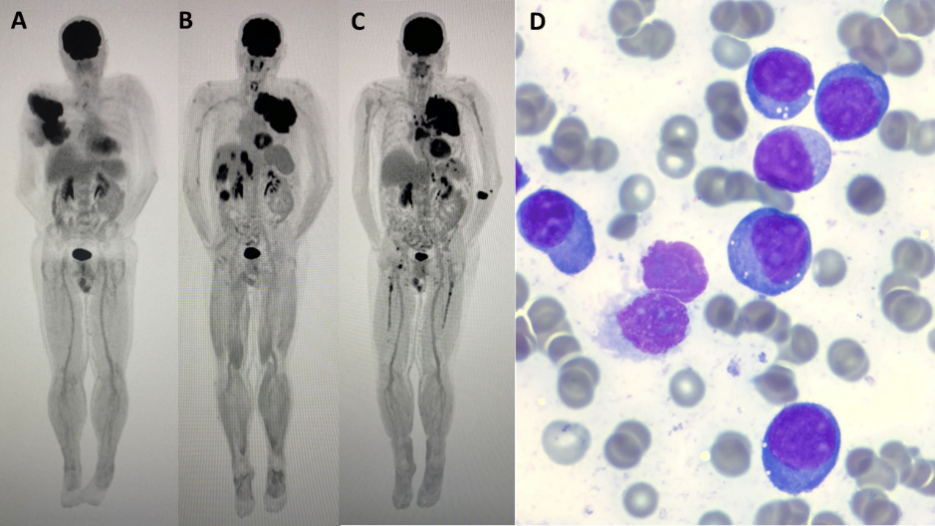
(A) PET/CT at diagnosis; (B) PET/CT at first relapse; (C) PET/CT at second relapse; (D) Bone marrow aspirate at second relapse (May-Grünwald-Giemsa stain x100 objective).

Plasmablastic myeloma is an aggressive variant of plasma cell neoplasm with poor survival.^1^ Plasmablastic myeloma is defined on bone marrow aspirate slides as containing ≥ 2% of plasmablasts.^2^ Plasmablasts are usually characterized as cells in which the cytoplasm has no or very little hof region, and less abundant cytoplasm (less than one-half of the nuclear area), large nucleus (diameter>10μm), fine chromatin pattern, and a large nucleolus >2μm.^1^ Plasmablastic morphology is an independent prognostic factor predicting shorter overall survival.^1^ Identifying this subtype is useful in practice. As the definition of plasmablastic myeloma may differ between studies, it is difficult to compare them and to apply a plasmablast classification in clinical trials. Here, we reported a case of secondary plasmablastic myeloma with 1q gain, 17p deletion, complex genome, and *BRAF* mutation. A novel treatment approach is warranted. BRAF-targeted therapy can be entertained.^3^ This case highlighted the importance of repeating imaging and biopsy at later relapse for risk stratification.

## Conflict of Interest disclosure statement

Authors have no conflict of interest
